# Diversity in susceptibility reactions of winter wheat genotypes to obligate pathogens under fluctuating climatic conditions

**DOI:** 10.1038/s41598-020-76693-z

**Published:** 2020-11-12

**Authors:** Radivoje Jevtić, Vesna Župunski, Mirjana Lalošević, Bojan Jocković, Branka Orbović, Sonja Ilin

**Affiliations:** Small Grains Department, Institute of Field and Vegetable Crops, Maksima Gorkog 30, 21000 Novi Sad, Serbia

**Keywords:** Plant sciences, Plant stress responses, Abiotic, Biotic

## Abstract

To date, studies have usually focused on the impact of abiotic factors on the distribution of plant pathogens and have built forecast models for the prediction of pathogen outbreaks. However, the impact of the combined effects of biotic and abiotic factors on the prevalence of economically important pathogens has usually been neglected. The objective of this study was to determine the relationship between powdery mildew and rusts of wheat and to examine how the combined effects of abiotic and biotic factors influence their prevalence. The study was conducted in the period 2016–2019 using the collection of 2158 genotypes of winter wheat. The most influential factors on disease indices and relationships among obligate pathogens were determined using multiple regression models and principal component analysis. The possibility of the coexistence of different rust species in the same growing season and in the same field was shown. The significant influence of fluctuations in winter temperatures on changes in the prevalence of obligate pathogens was determined. The strong impact of genotypes and their reaction on climatic elements in certain phenological stages were shown to be significant factors influencing the interactions among obligate pathogens and the predominance of one pathogen over another.

## Introduction

The impact of climate change on wheat production has become a global problem. It has been reported that a temperature increase of 1 °C would result in a decrease in wheat yields by 6%^1,2^. Moreover, alternations in climatic element patterns can cause changes in pathways of distribution and predominance of economically important pathogens^3^. The most important obligate pathogens of wheat are causal agents of powdery mildew (*Blumeria graminis* f. sp. *tritici*) and rust diseases (*Puccinia graminis* f. sp. *tritici*, *Puccinia triticina*, *Puccinia striiformis* f. sp. *tritici*). The reduction in winter wheat yields caused by powdery mildew could reach 45%^4^, and in Serbia, the yield loss estimates ranged from 17 to 40%^5^. Until 2014, the most common rust species in Serbia was leaf rust (*Puccinia triticina*), causing yield losses as high as 50%. However, due to changed climate conditions in 2014, yellow rust (*Puccinia striiformis* f. sp. *tritici*) predominated over leaf rust in Serbia^6^. In farmers’ fields, the disease severity of yellow rust in 2014 ranged from 40 to 60%, but in the genetic collection tested in the field trials of the Institute of Field and Vegetable Crops, Rimski Šančevi (Serbia), the disease indices of yellow rust reached 90% and resulted in a yield loss of 60%^7^.

Currently, pest control is challenging since wheat pathogens continuously change under selection pressures of agro-ecological conditions, resistant varieties and applied pesticides. Moreover, many efforts have been made to determine damage thresholds and develop mathematical models for forecasting wheat disease incidence and yield losses. Many of these studies have focused on the impact of climatic change on wheat yield and quality reduction by analysing the influence of abiotic factors^8–10^. However, the impact of the combined effects of biotic and abiotic factors on yield losses and disease occurrence has usually been neglected in studies^3,8^. Heeb^11^ promoted a strategy for climate-smart pest management (CSPM) but also noted that it is very unlikely that it would be possible to develop any general model for the prediction of climate change-induced pest outbreaks on a local scale in the short term.

Recently, it was reported that abiotic stressors, such as high- and low-temperature drought and salinity, could affect plant–pest interactions by altering plant physiology and defence responses^12^. The regulatory network for plant responses to abiotic and biotic stresses consists of many components that may function antagonistically, or some responses can be prioritized over others^13–15^. It was reported that stress combinations can have negative as well as positive effects on plants and that plant responses to combined stressors cannot be predicted by changes in response to individual stresses^12,13,16–18^. As a consequence, combinatorial stress needs to be treated and studied as a unique condition^12^, and the development of plants with enhanced tolerance to combined abiotic and biotic stresses should be prioritized.

Knowing that winter wheat genotypes could express different reactions due to fluctuating climatic elements, it was hypothesized in this study that the relationship among obligate pathogens of winter wheat is influenced by plant physiological responses to fluctuating climatic elements and that the occurrence of powdery mildew and rust diseases cannot be explained only on the basis of the influence of climatic elements on the life cycle of pathogens and the effects of genes determining plant resistance and pathogen virulence. Consequently**,** the objective of this study was to analyse the combined effect of abiotic and biotic factors on the prevalence of obligate pathogens in winter wheat, focusing on the reaction of susceptible genotypes (from moderately susceptible to very susceptible) on the combined effect of extreme fluctuation of climatic elements and obligate pathogen pressure. Data were obtained from a phenotyping platform for high-throughput screening of obligate pathogens (*B. graminis* f. sp. *tritici*, *P. graminis* f. sp. *tritici*, *P. triticina*, *P. striiformis* f. sp. *tritici*) in 2158 winter wheat genotypes with diverse genetic backgrounds. The results of this study provide broader insight into how plant–environment interactions affect the prevalence of powdery mildew and rust diseases in winter wheat, providing a basis for further studies on plant responses to combined stressors and ensuring the development of effective CSPM.

## Results

**Figure 1:**
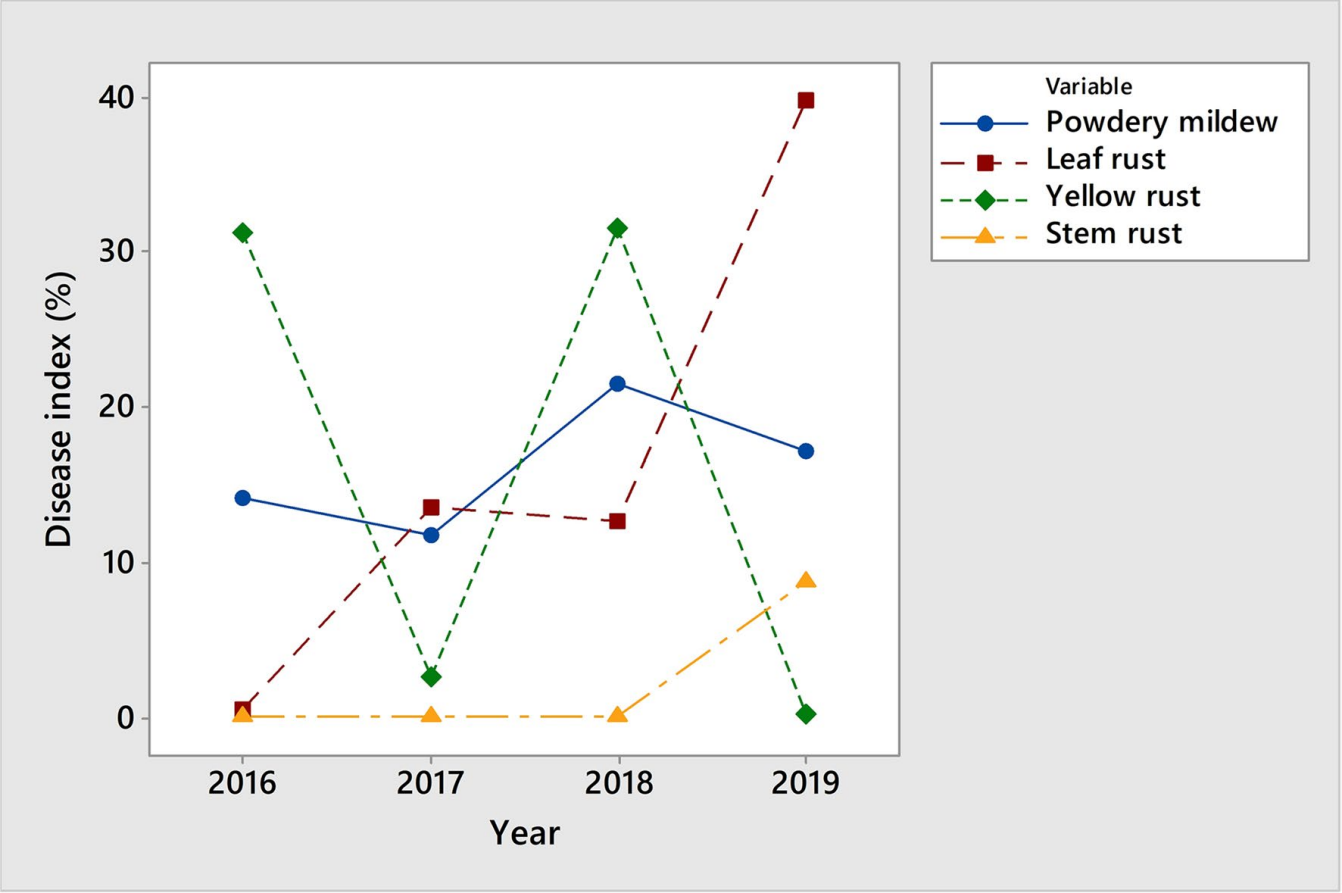
Average disease indices of powdery mildew and rust diseases on 2158 winter wheat genotypes in 2016–2019.

The relationship among obligate pathogens in the phenotyping platform was analysed on 2158 winter wheat genotypes for the period 2016–2019 (see Supplementary Dataset 1). The trend of occurrence and the relationship among powdery mildew and rust diseases in the phenotyping platform did not change linearly over the period 2016–2019 (Fig. 1). The disease indices of yellow rust in 2017 and 2019 had values near the trace level, but in 2016 and 2018, yellow rust was the prevalent pathogen, reaching average disease indices of 31.3% and 31.5%, respectively. The average disease indices of powdery mildew ranged from 11.7 to 21.5% in the whole period, indicating the consistent occurrence of powdery mildew regardless of the prevalence of different rust species. Leaf rust was the predominant rust species in the years that were not conducive to yellow rust occurrence. The average disease indices of leaf rust in the collection were 13.5% in 2017 and 39.8% in 2019. Stem rust outbreaks in the phenotyping site occurred in 2019, where 35% of genotypes showed symptoms of stem rust, with an average disease index of 24.8%. Disease indices above 25% were recorded for 16% of the genotypes. The average disease index of stem rust comprising susceptible (disease index above 30%) and resistant genotypes (disease index from trace level to 29%) was 8.7% (Fig. 1).

**Figure 2:**
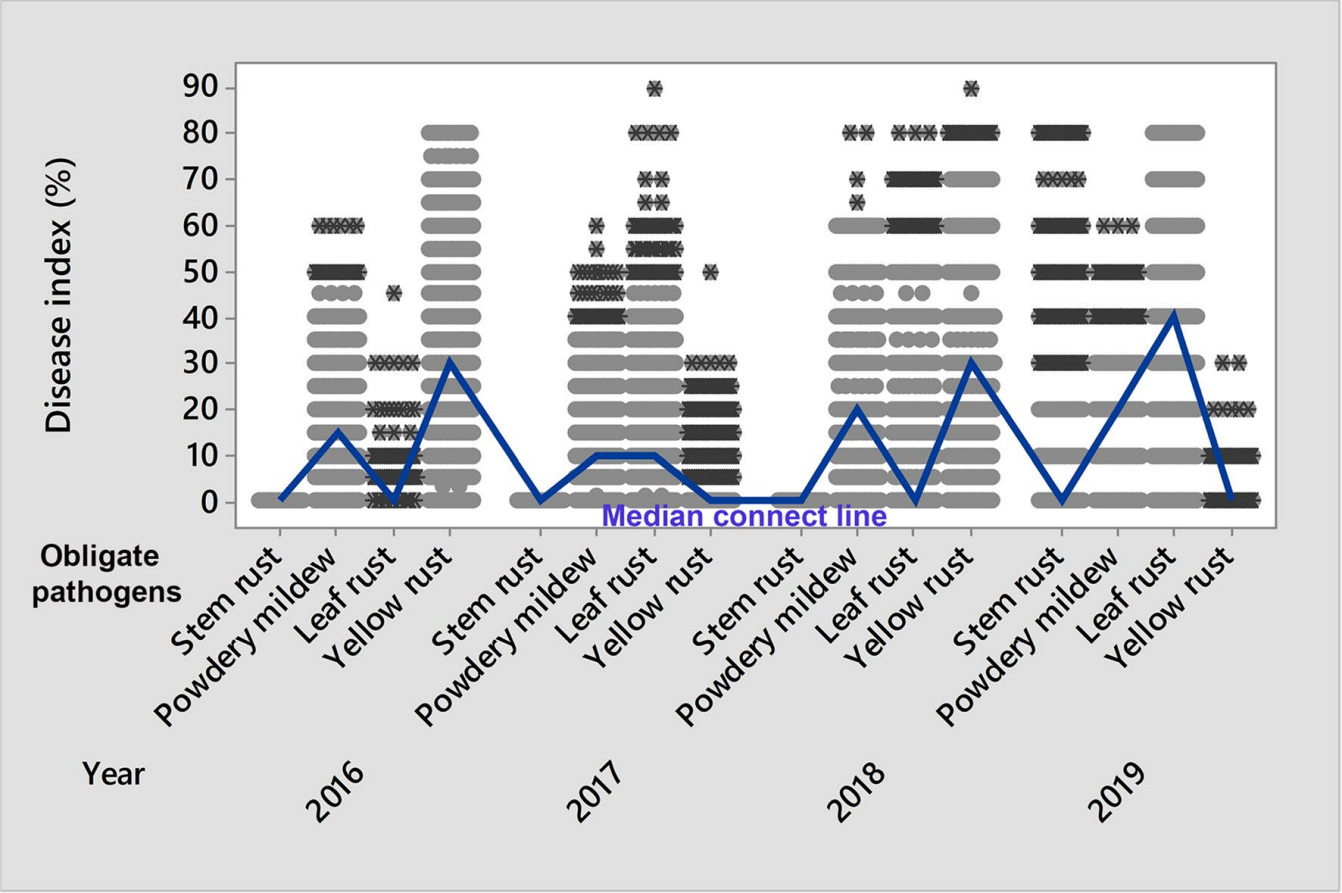
The range of disease indices of powdery mildew and rust diseases on 2158 winter wheat genotypes in 2016–2019.

The range of disease indices of each obligate pathogen in the phenotyping platform (Fig. 2) indicated great diversity in genotypes with respect to susceptibility/resistance to obligate pathogens. As a consequence, genotypes were recognized as influencing factors (*P* < 0.001) on disease indices of obligate pathogens using stepwise multiple regression. Moreover, obligate pathogens significantly influenced disease indices among each other (*P* < 0.001), indicating the necessity for a more detailed analysis of their interactions. The details of the multiple regression analysis are provided in Supplementary Table S1.

### Relationships among obligate pathogens in consecutive growing seasons

**Figure 3:**
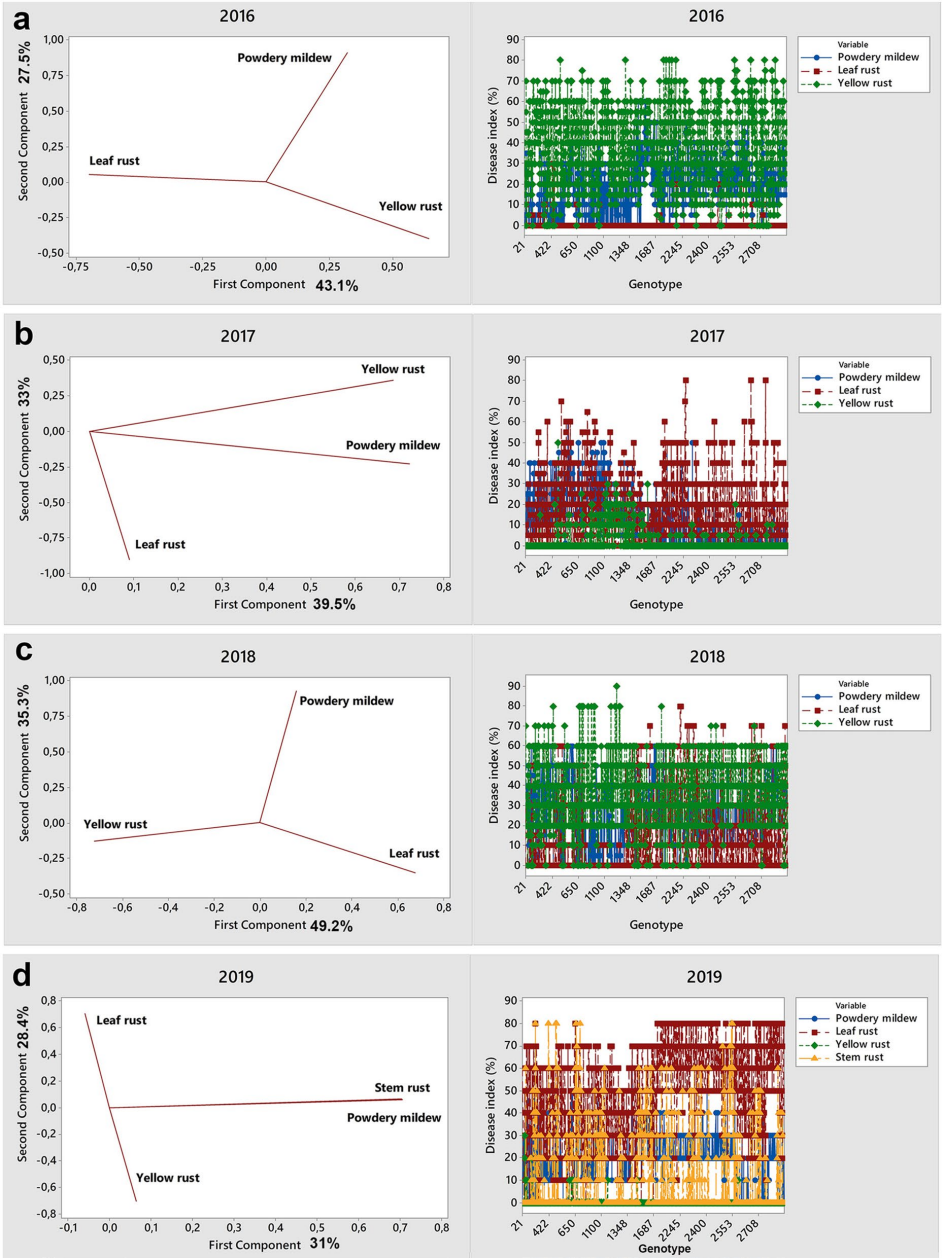
Relationship between powdery mildew and rust diseases on 1389 winter wheat genotypes in 2016–2019*: (**a**) PCA (left panel) and line plot (right panel) of disease indices of powdery mildew, yellow and leaf rust in the 2016 growing season; (**b**) PCA (left panel) and line plot (right panel) of disease indices of powdery mildew, yellow and leaf rust in the 2017 growing season; (**c**) PCA (left panel) and line plot (right panel) of disease indices of powdery mildew, yellow and leaf rust in the 2018 growing season; (**d**) PCA (left panel) and line plot (right panel) of disease indices of powdery mildew, yellow, leaf and stem rust in the 2019 growing season. *PCA was conducted on 1389 susceptible genotypes showing susceptibility to each obligate pathogen (from moderately susceptible to very susceptible) in at least one year in the period 2016–2019.

The relationships among obligate pathogens in each growing season were characterized for 1389 genotypes showing susceptibility to each obligate pathogen (disease index ˃ 30%) in at least one year in the period 2016–2019 (see Supplementary Dataset 2). Principal component analysis (PCA) was used to characterize the relationship between obligate pathogens in consecutive growing seasons by introducing disease indices of rusts of wheat and powdery mildew as input data (Fig. 3a–d).

According to PCA, leaf rust and yellow rust were usually negatively correlated, which was indicated by the angle of nearly 180° between them (Fig. 3a,c,d). These pathogens were a part of the first principal component in 2016 and 2018, which accounted for 43% and 49% of the total variance, respectively (Fig. 3a,c). In both years, the loading of leaf rust and yellow rust exceeded 0.50, indicating that both pathogens strongly influenced the first principal component. Interestingly, both years were conducive to yellow rust occurrence, but yellow rust and leaf rust influenced the first principal component with different value signs (Fig. 3a,c). Spearman’s correlation coefficients between yellow rust and leaf rust were in accordance with PCA (see Supplementary Table S2), indicating a statistically significant negative correlation (P < 0.001) in all growing seasons. However, the level of correlation ranged from low to moderate due to the diversity of susceptibility reactions of 1389 genotypes. Line plots (Fig. 3a–d) also showed a wide range of reactions of susceptible genotypes on each obligate pathogen in every growing season. According to PCA, powdery mildew was a part of the second principal component in 2016 and 2018, which accounted for 27.5% and 35.3% of the total variance, respectively. The angle between powdery mildew and rusts of wheat (leaf and yellow rust) was usually nearly 90°, indicating no correlation between these pathogens. The exception was 2017, when powdery mildew and yellow rust were positively correlated. Similar to PCA, Spearman’s coefficient showed no correlation between powdery mildew and rusts of wheat (see Supplementary Table S2), except in 2017, when powdery mildew was moderately correlated with yellow rust (r = 0.29, *P* ˂ 0.001**)**. Regarding PCA, in 2017 and 2019, the relationships among obligate pathogens were different than those in 2016 and 2018. In 2017, powdery mildew and yellow rust were part of the first principal component, while in 2019, powdery mildew and stem rust took part in the first principal component. According to Spearman’s correlation coefficients, stem rust did not correlate with any obligate pathogen except for powdery mildew, which showed a moderate positive correlation (r = 0.28, *P* ˂ 0.001**)** (see Supplementary Table S2). Leaf rust was part of the secondary principal component in both 2017 and 2019 (Fig. 3b,d).

These results indicated that the relationship among obligate pathogens is not straightforward and is strongly influenced by the growing season and reaction of genotypes on fluctuating climatic elements. As a consequence, a detailed analysis of the combined effect of abiotic and biotic factors on the prevalence of obligate pathogens in winter wheat was conducted on different sets of susceptible genotypes (from moderately susceptible to very susceptible) in growing seasons that were conducive to the occurrence of obligate pathogens of interest.

### Inconsistency in the predominance of powdery mildew and yellow rust in the same field and growing season

**Figure 4:**
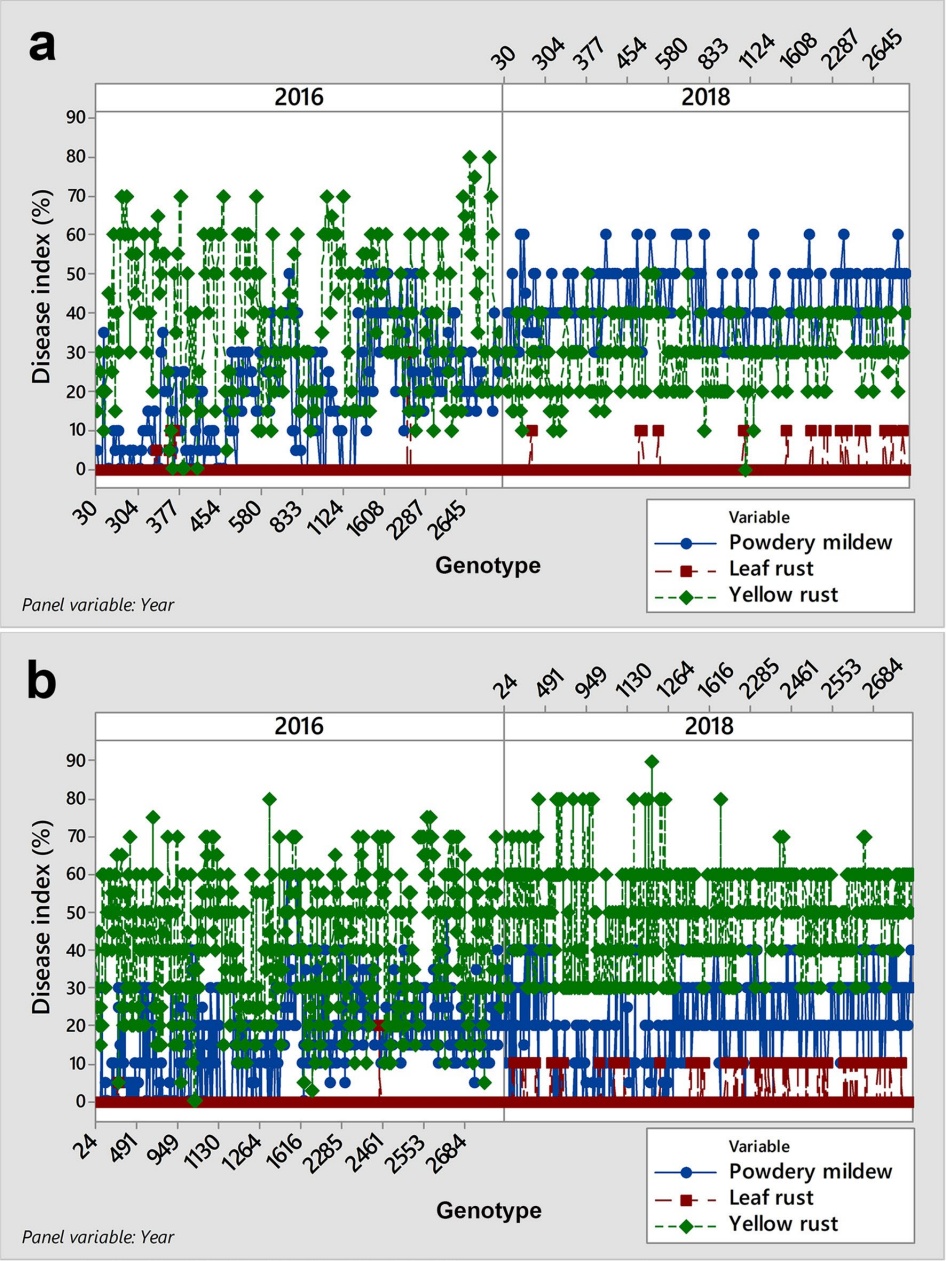
Line plots showing the relationship between powdery mildew and yellow rust diseases on 740 winter wheat genotypes in 2016 and 2018*: (**a**) set of genotypes where powdery mildew predominated over yellow rust in 2018; (**b**) set of genotypes where yellow rust predominated over powdery mildew in 2018. In both sets of genotypes, yellow rust predominated over powdery mildew in 2016. *Genotypes included in analysis were those where disease indices of leaf rust did not exceed 10%.

As shown above (Fig. 1), the average disease indices of powdery mildew ranged from 11.7 to 21.5% and were present in each growing season regardless of the prevalence of different rust species. In 2018, among 1989 genotypes showing symptoms of yellow rust, the predominance of yellow rust over powdery mildew occurred in 55.5% of genotypes, while in the same growing season and in the same field, powdery mildew predominated over yellow rust in 33% of genotypes. To determine which factors influenced diversity in genotype responses to powdery mildew and yellow rust in 2018, multiple linear regression was conducted on 740 genotypes where disease indices of leaf rust did not exceed 10% in both 2016 and 2018; thus, the relationship between powdery mildew and yellow rust was estimated without interference from other obligate pathogens (see Supplementary Dataset 3). In addition, 740 genotypes were divided into two sets: (1) powdery mildew predominated over yellow rust in 2018 (Fig. 4a), and (2) yellow rust predominated over powdery mildew in 2018 (Fig. 4b). Since yellow rust predominated over powdery mildew in both sets of genotypes in 2016 (Fig. 4a,b), climatic factors related to both growing seasons (2015/2016 and 2017/2018) were subjected to multiple linear regression together with the effect of genotypes and competing obligate pathogens to investigate the most influential factors on the disease indices of yellow rust and powdery mildew in the two sets of genotypes. Climatic conditions included in statistical analysis were monthly mean temperatures, relative humidity and total rainfall taken for entire growing seasons. Since stepwise regression was applied, only those variables recognized as significant were retained in the table of variance analysis (see Supplementary Table S3).

Multiple regression analysis indicated that the differences in temperatures in January and February in 2016 and 2018 resulted in different reactions of the two sets of genotypes on powdery mildew and yellow rust. In the set of genotypes where yellow rust predominated over powdery mildew, the temperature in January (*P* ˂ 0.001) and genotypes (*P* ˂ 0.001) were recognized as the most influencing factors on the disease indices of yellow rust and powdery mildew, with coefficients of determination (R^2^) of 61% and 63%, respectively. The temperature in January of 4.3 °C in 2018 exceeded the long-term average of − 0.1 °C and provided preconditions for yellow rust occurrence, but yellow rust did not predominate over powdery mildew in all analysed genotypes. There was a set of genotypes where powdery mildew predominated over yellow rust, and the most influential factors on the disease indices of powdery mildew were temperature in February (*P* ˂ 0.001), yellow rust (*P* = 0.028) and genotype (*P* = 0.023), with an R^2^ of 77.8%. The temperature in February (1.2 °C) in 2018 was nearly 7 °C below the temperature in February in 2016 (7.5 °C), providing preconditions for the predominance of powdery mildew over yellow rust in that year, which was initially favourable for yellow rust occurrence. An analysis of residuals and the test for the possible occurrence of outliers showed that there were no outliers in the data. The normal probability plot showed an approximately linear pattern, which was consistent with a normal distribution (see Supplementary Fig. S1).

These results indicated that the relationship between yellow rust and powdery mildew was strongly influenced by both climatic elements and the reaction of genotypes to fluctuations in climatic elements and that the reaction of wheat genotypes to extreme fluctuations in winter climatic elements could influence the relationship between powdery mildew and yellow rust, favouring powdery mildew over yellow rust.

### Inconsistency in the predominance of leaf rust and yellow rust in the same field and growing season

As mentioned above, 2016 was very conducive to yellow rust occurrence, which was in contrast to 2017, when a temperature of − 5 °C in January generally caused a lower level of infection with yellow rust. In 2018, the average temperature in January (4.2 °C) exceeded the long-term average, favouring yellow rust occurrence. Among 1989 genotypes with symptoms of rust diseases, yellow rust predominated over leaf rust in 59% of genotypes. However, the predominance of leaf rust over yellow rust was also recorded on 21% of genotypes in the same year and the same field. Knowing that yellow rust is a more aggressive pathogen, the predominance of leaf rust over yellow rust drew our attention.

**Figure 5:**
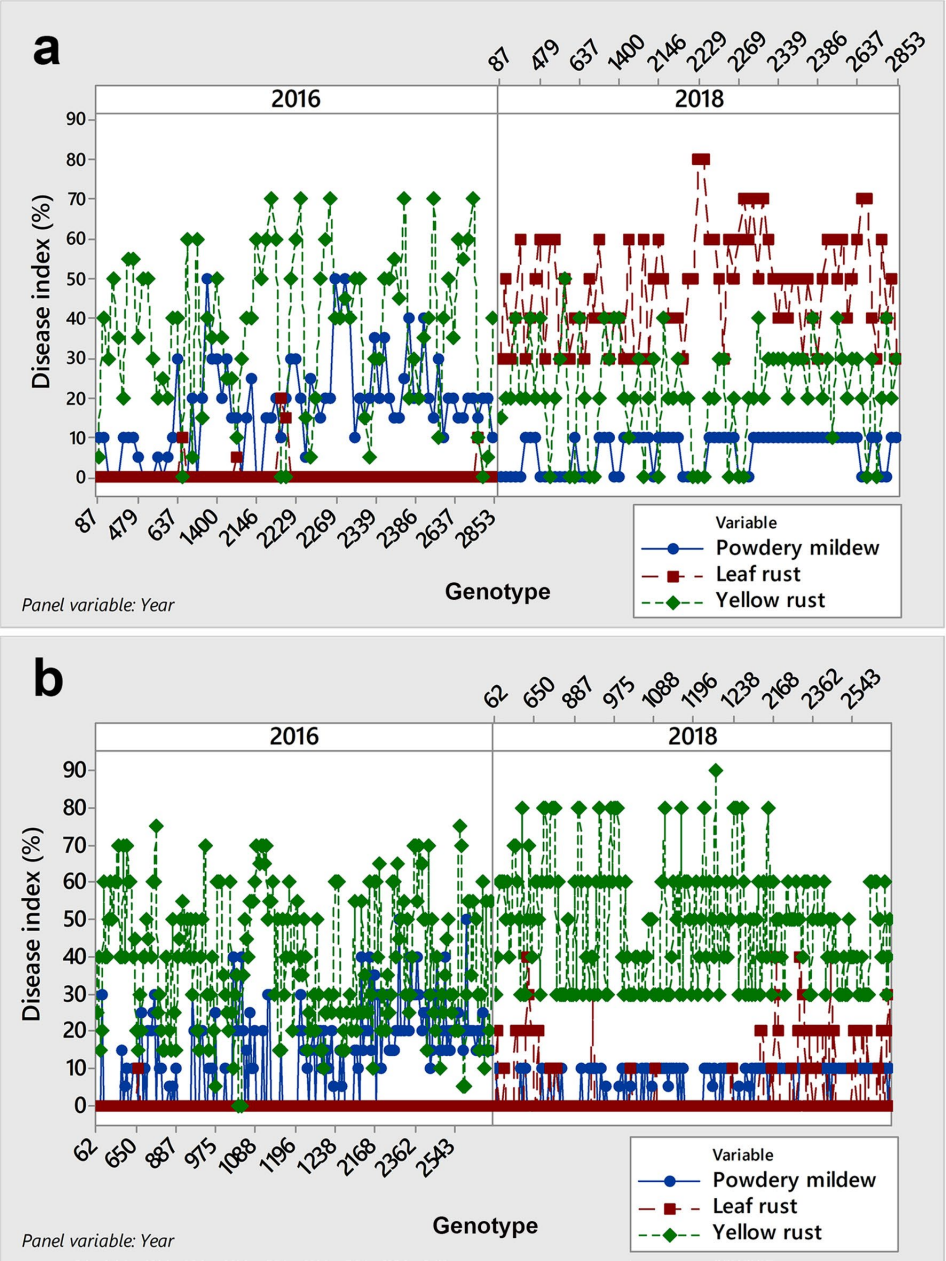
Line plots showing the relationship between leaf and yellow rust diseases on 303 winter wheat genotypes in 2016 and 2018*: (**a**) set of genotypes where leaf rust predominated over yellow rust in 2018; (**b**) set of genotypes where yellow rust predominated over leaf rust in 2018 In both sets of genotypes, yellow rust predominated leaf rust in 2016. *Genotypes included in analysis were those where disease indices of powdery mildew did not exceed 10%.

To determine which factors influenced diversity in genotype responses on leaf and yellow rust in 2018, multiple linear regression was conducted on 303 genotypes where disease indices of powdery mildew did not exceed 10% in both 2016 and 2018; thus, the relationship between leaf and yellow rust was estimated without interference from other obligate pathogens (see Supplementary Dataset 4). In addition, 303 genotypes were divided into two sets: (1) leaf rust predominated over yellow rust in 2018 (Fig. 5a), and (2) yellow rust predominated over leaf rust in 2018 (Fig. 5b). Since yellow rust predominated over leaf rust in both sets of genotypes in 2016 (Fig. 5a,b), climatic factors related to both growing seasons (2015/2016 and 2017/2018) were subjected to multiple linear regression together with the effect of genotypes and competing obligate pathogens to investigate the most influencing factors on disease indices of leaf and yellow rust in two sets of genotypes. The climatic conditions included in statistical analysis were monthly mean temperatures, relative humidity and total rainfall taken for entire growing seasons. Since stepwise regression was applied, only those variables recognized as significant were retained in the table of variance analysis (see Supplementary Table S4).

After a more detailed analysis of the factors influencing the relationship between leaf and yellow rust using multiple linear regression in two growing seasons, it was shown that the influence of genotype × environment interaction should not be understated in studies related to yellow rust and leaf rust occurrence. Temperature in February (*P* = 0.001) and yellow rust (*P* ˂ 0.001) were recognized as influencing factors on disease indices of leaf rust in the set of genotypes where leaf rust predominated over yellow rust, with an R^2^ of 86%. Interestingly, the same result was obtained in the set of genotypes where powdery mildew predominated over yellow rust. In the set of genotypes where yellow rust predominated over leaf rust, temperature in January (*P* ˂ 0.001) was equally the most influencing factor on the occurrence of both pathogens, as it was in the set of genotypes where yellow rust predominated over powdery mildew. The results of this study showed that the interaction between yellow rust and leaf rust is highly influenced by the response of wheat genotypes to fluctuating climate elements and that leaf rust could predominate over yellow rust, although yellow rust was considered a more aggressive pathogen.

### Stem rust: a hidden threat to wheat production

Breeding for rust resistance and monitoring the distribution of rust races are the main tools used to control rust pathogens and maintain sustainable wheat production. However, the question of how fluctuating climate elements and shifts in the duration of wheat phenological phases could impact the prevalence of obligate pathogens has yet to be investigated.

In the 2018/2019 growing season, Serbia was facing an extreme reduction in total rainfall at the time of sowing (October), which continued through November and caused a delay in the emergence of winter wheat (see Supplementary Fig. S2). The total rainfall in October was 7.4 mm, which was six times lower than the long-term average of 47.2 mm dating back to 1964. The same trend continued in November with a total rainfall of 24.6 mm, which was also lower than the long-term average of 46.2 mm. Reduction of total rainfall in February and March, which is the construction phase (see Supplementary Fig. S2) caused delays in stem elongation and booting of winter wheat genotypes and provided preconditions for stem rust outbreaks in the phenotyping platform in 2019. In addition to stem rust, leaf rust and powdery mildew were recorded in the collection in 2019. In agro-ecological conditions in Serbia, precipitation in November (*P* < 0.001) together with leaf rust (*P* = 0.007) and powdery mildew (*P* = 0.001) were recognized as influencing factors on the infection level of stem rust in 2019, with an R^2^ of 51.4%. The details of the multiple regression analysis are provided in (see Supplementary Table S5).

**Figure 6:**
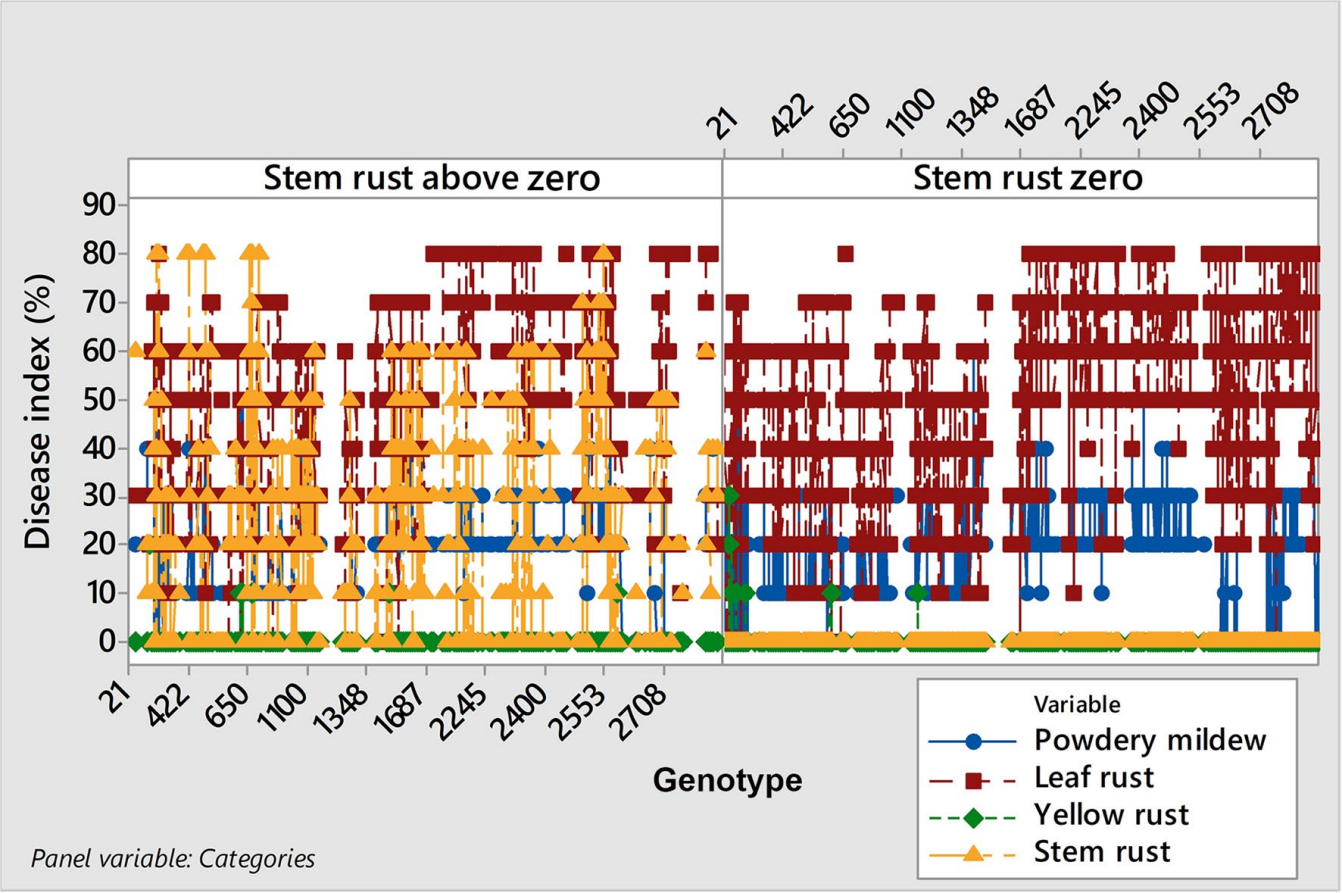
Occurrence of stem rust in the phenotyping platform in 2019 and its relationship with powdery mildew, leaf and yellow rust.

In 2019, stem rust occurred on 35% of all genotypes in the collection (see Supplementary Dataset 5). The disease indices of stem rust ranged from trace levels to 80%. Leaf rust and powdery mildew were present to the same extent in both groups (infected and non-infected with stem rust), indicating the coexistence of stem rust with powdery mildew and leaf rust (Fig. 6).

A significantly low positive correlation (r = 0.28, *P* ˂ 0.001) between stem rust and powdery mildew followed by a low R^2^ of 5.7% as well as a lack of correlation between stem and leaf rust indicated that those pathogens could coexist in the same host plant and in the same field.

The significant influence of total rainfall in November on the disease indices of stem rust indicated that the delay of phenological phases due to drought in the early phases of winter wheat development made 35% of genotypes more vulnerable to stem rust infection. The low coefficient of correlation between stem rust and powdery mildew and the lack of correlation between stem rust and leaf rust showed the possibility of the co-expression of these pathogens, which can be partly explained by the invasion of different plant organs. However, powdery mildew and leaf rust were also recognized as factors influencing the level of stem rust infection, indicating that more investigations should be done to explain the nature of their interaction.

## Discussion

In this study, the relationship between rust species and powdery mildew was not straightforward over the study period. Climatic conditions were basic precursors for pathogen infection and disease development in each growing season, but the strong impact of genotypes and their reaction on climatic elements were shown to be significant factors influencing the interaction among obligate pathogens and the predominance of one pathogen over the other.

Powdery mildew was present in the phenotyping platform in each growing season regardless of the prevalence of different rust species. However, in 2018, powdery mildew predominated over yellow rust in 33% of genotypes, and fluctuation in winter temperatures was recognized as a significant factor influencing the relationship between these two pathogens. It was reported that the expression of resistant genes to powdery mildew is temperature dependent and that both low and high temperatures could influence their effectiveness^19^. However, this study indicated that the reaction of wheat genotypes to extreme fluctuations in winter climatic elements influenced the relationships among obligate pathogens on susceptible genotypes as well.

Yellow rust and leaf rust are known to have different requirements for climatic conditions for the initiation of infection and disease development^20^. It was reported that yellow rust would predominate over leaf rust if requirements for high winter temperatures were met^21^. Specific requirements of yellow rust for high winter temperatures initiated the development of yellow rust forecasting models in many countries^22,23^. Those models usually consisted of different combinations of winter climatic elements depending on region and climate zone, but the impact of cultivars on yellow rust occurrence was usually underestimated and excluded from yellow rust forecasts. In this study, in 2018, leaf rust predominated over yellow rust in 21% of genotypes, although yellow rust is considered a more aggressive pathogen. Diane Saunders from John Innes Centre, UK, proved that the Warrior Race of wheat yellow rust became predominant in Serbia, as in many European countries^6,21^. In Serbia, yellow rust predominated leaf rust in 2014 when winter temperatures in January (4.2 °C) and February (6.1 °C) exceeded the long-term averages of -0.1 °C in January and 1.8 °C in February, based on the record dating back to 1964^6^. In contrast, in this study, during the period 2016–2019, high winter temperatures were assessed in different months, resulting in an inconsistency in the reaction of susceptible genotypes to obligate pathogens. The screening of the population structure of yellow rust during the period 2016–2019 was also conducted in cooperation with Dr. Diane Saunders, and it was proven that there were no changes in the population structure of yellow rust in the examined locality compared with that in 2014 (personal communication).

It was reported that the reaction of plants to combined stressors cannot be predicted, and in this study, the reaction of genotype to fluctuating winter temperatures favoured the predominance of obligate pathogens against yellow rust in the years that were initially conductive to yellow rust occurrence. It was the case not only for powdery mildew whose first symptoms in agro-ecological conditions in Serbia could be recorded in February but also for leaf rust whose optimal conditions for initiation of infection are usually met in the first half of April^6^. The average temperature in April 2018 (17.2 °C) favoured leaf rust occurrence and was higher than that in 2016, 2017 and 2019. However, the temperature in April in the period 2016–2019 was also in accordance with the temperature range of 12–15 °C reported to be optimal for the germination of leaf rust urediniospores^24^, which is a reason why the temperature in April was not recognized as an influential factor on the different reactions of winter wheat genotypes to leaf rust and yellow rust infection in 2018.

The field trials in this study support some investigations conducted in controlled conditions where plant responses to obligate pathogens were either independent of resistance genes and determined with temperature changes^25^ or influenced by calcium-dependent phospholipid-binding proteins responsive to both pathogen infection and temperature changes^26^. The results of this study showed that in agro-ecological conditions in Serbia, high temperatures in February could initiate a predominance of yellow rust in a greater number of winter wheat genotypes compared to the number of genotypes in the year when high winter temperatures are met only in January. Consequently, the results of this study indicated that more attention should be focused on the mechanisms by which winter wheat genotypes could escape severe infections with yellow rust in the years that were initially conductive to the development of this pathogen.

Stem rust was well controlled in many parts of the world until 1998, when it drew great attention due to the outbreak of the Ug99 race in Uganda^27^. Stem rust race Ug99 is known to be virulent to 80% of wheat varieties in the world^27^. In addition to the Ug99 race, stem rust race TTTTF caused resistance breakdown of many durum and bread wheat varieties in Sicily in 2016 and was reported to be a serious threat for other European countries. Stem rust was a common wheat pathogen in Serbia in the 1960s, causing yield losses up to 31%, but with the introduction of early winter wheat varieties, it was reduced or almost eradicated^28^. In 2017, the TTTTF race was recorded in a localized area of Rimski Šančevi on genotypes that were planted in the spring and tested for photoperiodism reactions^28^. This finding drew attention to the threat of stem rust re-establishment in Serbia^28^. Following this study, stem rust outbreaks in the phenotyping platform occurred again in 2019 when 35% of genotypes that had previously been able to avoid inoculum build-up were infected with stem rust due to drought in the early phases of wheat development. The significant influence of total rainfall in November on the disease indices of stem rust indicated that delayed development of winter wheat genotypes due to extreme fluctuations in total rainfall in the winter period can make winter wheat genotypes more vulnerable to stem rust infection.

## Conclusions

Serbia is facing extreme fluctuations in climatic elements, as has been reported in other parts of the world. In the last four years, temperatures in January and February differed in the range of almost 10 °C (from − 5 to + 5 °C in January) and 7 °C (from 1.2 to 7.5 °C in February). The difference in the total rainfall range of 24.6–67.1 mm in November and 18–81 mm in February was also recorded. The combined effect of abiotic and biotic factors on powdery mildew rust disease occurrence has usually been neglected in studies. This study noted that more attention should be focused on the combinatorial stress responses of plants that consequently influence the distribution, prevalence and management of economically important pathogens. It was shown in this study that fluctuation of winter temperatures in early phases of wheat development could result in differences in the reaction of susceptible winter wheat genotypes to obligate pathogens, making forecasts of disease prevalence impossible. This finding indicated that combined stresses needs to be treated as a unique condition to ensure the development of effective CSPM.

Many studies have shown that altering climatic element patterns can cause changes in pathways of distribution and predominance of economically important pathogens. However, understanding the mechanisms by which climatic elements influence pathogen/host interactions would provide new insights into many aspects of integrated pest management and wheat production worldwide.

## Methods

### Field trials

Data were obtained from a phenotyping platform for high-throughput screening of obligate pathogens (*B. graminis* f. sp. *tritici*, *P. graminis* f. sp. *tritici*, *P. triticina*, *P. striiformis* f. sp. *tritici*) in 2158 winter wheat genotypes with diverse genetic backgrounds (see Supplementary Dataset 1). Evaluation of winter wheat genotypes for resistance to obligate pathogens was conducted in the locality of Rimski Šančevi (Vojvodina, northern province of Serbia) under the direction of the Institute of Field and Vegetable Crops, Novi Sad, Serbia, over the period of 2016 to 2019. Screenings of winter wheat genotypes were a part of pre-breeding and breeding efforts of breeding programmes in developing and promoting wheat lines with resistance to rust diseases and powdery mildew.

Field trials were set up using methodology recommended by CIMMYT in the “Instructions for the management and reporting of results for all International Screening Nurseries: Bread wheat (IBWSN), Durum (IDSN), Triticale (ITSN), and Septoria (ISEPTON)”^29^. This methodology was designed for the rapid assessment of a large number of advanced generation (F3–F7) lines of wheat genotypes under a wide range of climatic and disease conditions. According to this methodology, each genotype should be sown in one unreplicated 5-m row or using smaller row lengths but with replicates so that the sum of all row lengths is equal to 5 m. In our study, field trials were set up under naturally occurring inoculum, and each genotype was sown in 1-m rows in five replicates with row spacing of 20 cm, giving a plot area of 1 m^2^. The soft wheat variety Barbee (*Triticum aestivum* ssp. *compactum*) that showing increased susceptibility to obligate parasites (*Blumeria* and *Puccinia*) was planted in replicate across the phenotyping platform to control and ensure constant pathogen pressure on all tested genotypes. Assessments of obligate pathogens were expressed with disease indices (DIs, %) and made at the growth stage 71–73 BBCH^30^ (kernel watery; early milk), known to be highly related to yield^31^. The DIs (%) of rusts and powdery mildew were calculated as a product of disease incidence (mean % plants infected per plot) and average disease severity^32^. Disease severity is defined as the percentage of relevant host tissues or organs covered by symptoms^32^ and was estimated on entities within a sampling unit using modified Cobb’s scale^33,34^. Consequently, DI was calculated as follows: DI (%) = [sum (class frequency × score of rating class)]/[(total number of plants) × (maximal score of rating class)] × 100. The mean sowing date for winter wheat was 20 October (optimal time of sowing) in agro-ecological conditions in Serbia. Because the level of susceptibility or resistance of the genotypes highly influenced the occurrence of the pathogens, the interaction among powdery mildew and rust diseases was analysed for 1389 susceptible genotypes showing susceptibility to each obligate pathogen (from moderately susceptible to very susceptible) in at least one year in the period 2016–2019 (see Supplementary Dataset 2). The level of susceptibility was determined as follows: 0–5—very resistant; 6–10%—resistant; 11–29%—moderately resistant; 30–40%—moderately susceptible; 41–65%—susceptible; and > 66%—very susceptible.

The consistency of the population structure of rust species at the phenotyping site was monitored in collaboration with Dr. Diane Saunders from John Innes Centre, UK. The predominance of Warrior races of yellow and TTTTF races of stem rust was confirmed (personal communication).

### Statistical methods

The relationship among obligate pathogens in each growing season was characterized using principal component analysis (PCA) by introducing disease indices of rusts and powdery mildew as input data. The approximation of the correlation between the variables was made based on the angle between the vectors. A small angle indicates that the variables are positively correlated, an angle of 90 degrees indicates that the variables are not correlated, and an angle close to 180 degrees indicates that the variables are negatively correlated. Since PCA does not quantify the relationship between variables, the relationship between disease indices of rusts of wheat and powdery mildew per growing season was characterized using Spearman’s coefficient of correlation.

The factors influencing the disease indices of rusts of wheat and powdery mildew (dependent variables) were analysed using a multiple linear regression model (stepwise regression). Genotypes were considered categorical variables in multiple linear regression models. Since different species of obligate pathogens can be habitant of the same host plant, obligate pathogens and climatic elements (temperature, relative humidity, and total rainfall) were referred to as continuous variables. Monthly mean temperatures, relative humidity and total rainfall were taken for each growing season (from November to June) using data from the Republic Hydrometeorological Service of Serbia (https://www.hidmet.gov.rs/). Due to the nature of the data, the transformation $\sqrt {x + 0.5}$ was performed. Transformation $\sqrt {x + 0.5}$ is usually performed when a variable has one or more zeros^35^.

Knowing that a complete set of variables in the regression model would give larger variance in the regression coefficients, subsets of variables were used for building linear regression models. Since abiotic and biotic factors could be correlated (multicollinearity), which makes the evaluation of the individual impact of each of the correlated predictors on the response variable difficult, stepwise regression was applied. In stepwise regression, the most significant variables were systematically added, and the least significant variables were systematically removed during each step of the process until it identified variables that explained the maximum variation in the dependent variable. Alpha to enter and alpha to remove the influencing factors in stepwise multiple regression was set by default to be 0.15 since it was reported that level such as 0.05 can fail in identifying variables known to be important^36^. Multicollinearity was checked in all regression analyses and estimated thorough VIF values. A VIF between 5 and 10 indicates a high correlation that may be problematic, but in our study, it never exceeded a value of 5. Coding of categorical predictors was performed by choosing (− 1, 0, + 1) to estimate the difference between each level mean and the overall mean.

Regression models were followed with the coefficient of determination (R^2^), which is the percentage of variation in the response that is explained by the model. The correlation between levels of infection among obligate pathogens was estimated using Spearman’s coefficient of correlation. The entire analysis was performed using Minitab 17 (trial version).

## Supplementary information

Supplementary Dataset 1.

Supplementary Dataset 2.

Supplementary Dataset 3.

Supplementary Dataset 4.

Supplementary Dataset 5.

Supplementary Figures.

Supplementary Tables.

Supplementary Captions.

## Data Availability

The datasets generated during the current study are available from the corresponding author upon reasonable request.
