# Supplementary material for: Diversity in susceptibility reactions of winter wheat genotypes to obligate pathogens under fluctuating climatic conditions
Source: Sci Rep. 2020 Nov 12;10:19608. doi: 10.1038/s41598-020-76693-z (PMC7665191; doi:10.1038/s41598-020-76693-z)
Supplement: Supplementary file 6 — Supplementary Figures. [file 41598_2020_76693_MOESM6_ESM.pdf]

Supplementary information
for the manuscript:

Diversity in susceptibility reactions of winter wheat genotypes to obligate pathogens under fluctuating climatic conditions

Radivoje Jevtić[1*], Vesna Župunski[1], Mirjana Lalošević[1], Bojan Jocković[1], Branka Orbović[1], Sonja Ilin[1]

[1] Institute of Field and Vegetable Crops; Small Grains Department; Maksima Gorkog 30, 21000 Novi Sad, Serbia
* Correspondence: radivoje.jevtic@ifvcns.ns.ac.rs; Tel.: +381-21-4898207; Fax: +381-21-4898222

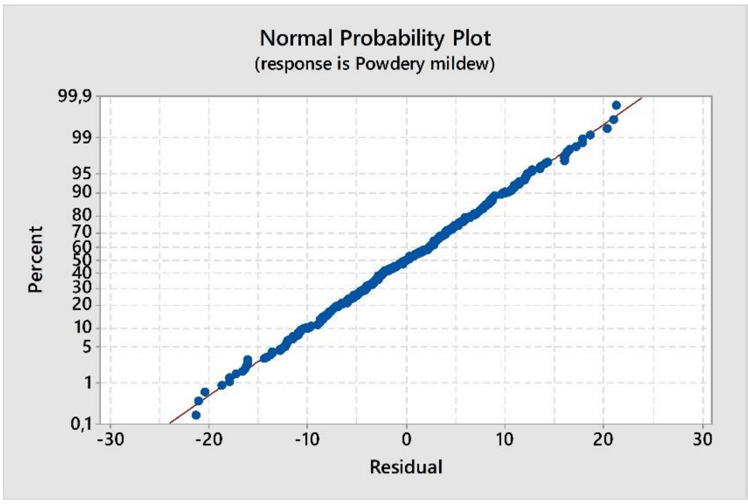

Supplementary Figure S1. Residual analysis of multiple regression on the factors influencing disease indices of powdery mildew in the set of genotypes where yellow rust predominated powdery mildew in 2016 and powdery mildew predominated yellow rust in 2018

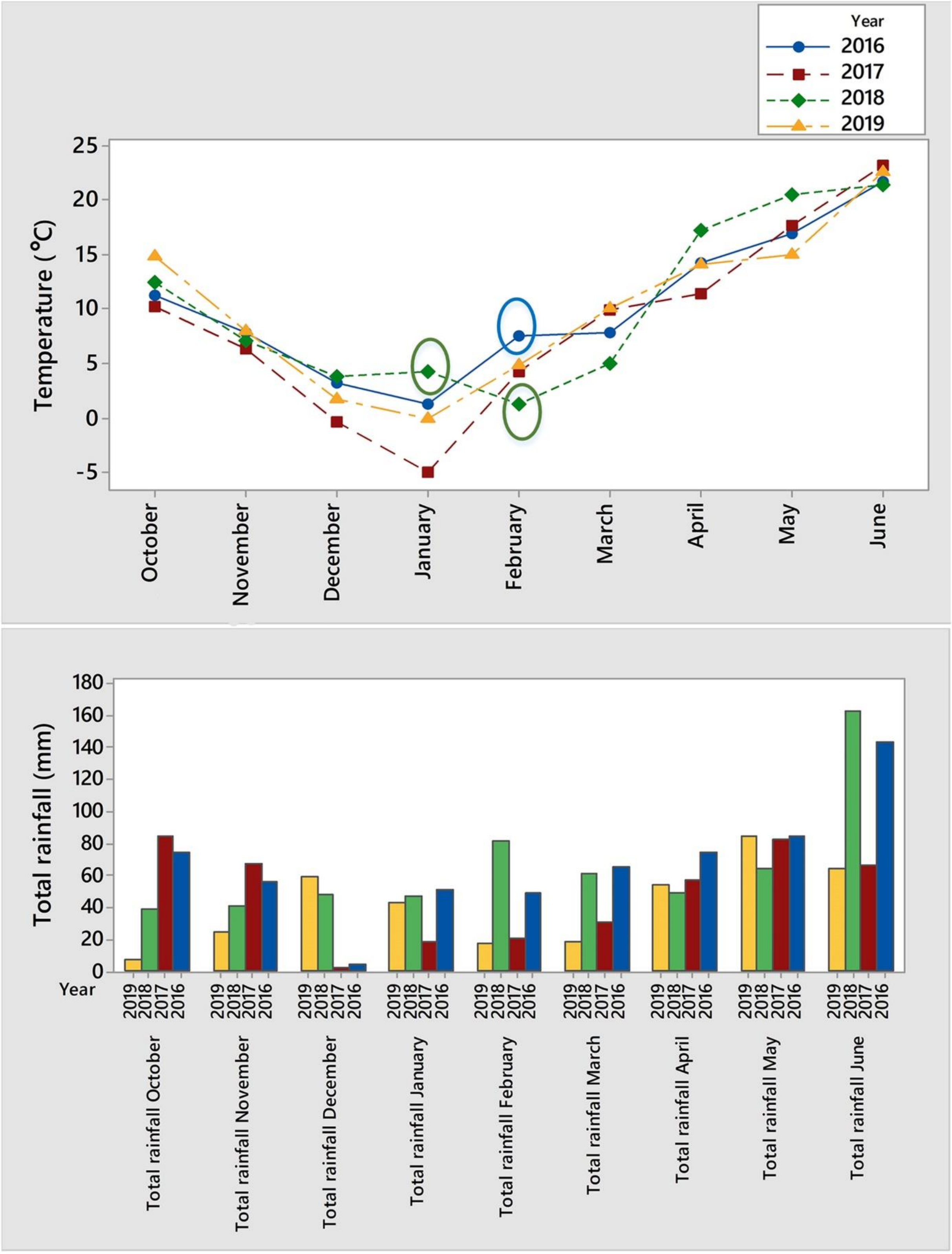

Supplementary Figure S2. Average temperatures and total rainfall in locality Rimski Šančevi in the period 2016-2019.
