# Supplementary material for: Diversity in susceptibility reactions of winter wheat genotypes to obligate pathogens under fluctuating climatic conditions
Source: Sci Rep. 2020 Nov 12;10:19608. doi: 10.1038/s41598-020-76693-z (PMC7665191; doi:10.1038/s41598-020-76693-z)
Supplement: Supplementary file 7 — Supplementary Tables. [file 41598_2020_76693_MOESM7_ESM.pdf]

Supplementary information
for the manuscript:

Diversity in susceptibility reactions of winter wheat genotypes to obligate pathogens under fluctuating climatic conditions

Radivoje Jevtić[1*], Vesna Župunski[1], Mirjana Lalošević[1], Bojan Jocković[1], Branka Orbović[1], Sonja Ilin[1]

[1] Institute of Field and Vegetable Crops; Small Grains Department; Maksima Gorkog 30, 21000 Novi Sad, Serbia
* Correspondence: radivoje.jevtic@ifvcns.ns.ac.rs; Tel.: +381-21-4898207; Fax: +381-21-4898222

Supplementary Table S1. Regression analysis of the most influencing factors on disease indices of obligate pathogens in 2158 winter wheat genotypes for the period 2016-2019

**Depenedent variable: disease index of yellow rust**					
Source	DF	Adj SS	Adj MS	F-Value	P-Value
Regression	2163	48183,3	22,28	11,37	0,000
Stem rust	1	77,6	77,60	39,60	0,000
Leaf rust	1	604,9	604,88	308,72	0,000
Powdery mildew	1	282,8	282,84	144,36	0,000
Year	3	18728,3	6242,78	3186,22	0,000
Genoypte	2157	7965,8	3,69	1,88	0,000
Error	6462	12661,0	1,96		
Total	8625	60844,3			
**Depenedent variable: disease index of powdery mildew**					
Source	DF	Adj SS	Adj MS	F-Value	P-Value
Regression	2163	13283,8	6,141	2,10	0,000
Stem rust	1	173,4	173,442	59,18	0,000
Leaf rust	1	30,3	30,323	10,35	0,001
Yellow rust	1	423,1	423,062	144,36	0,000
Year	3	781,7	260,565	88,91	0,000
Genoypte	2157	10357,2	4,802	1,64	0,000
Error	6462	18937,7	2,931		
Total	8625	32221,5			
**Depenedent variable: disease index of leaf rust**					
Source	DF	Adj SS	Adj MS	F-Value	P-Value
Regression	2163	43479,5	20,10	6,63	0,000
Stem rust	1	85,5	85,48	28,19	0,000
Powdery mildew	1	31,4	31,37	10,35	0,001
Yellow rust	1	936,1	936,05	308,72	0,000
Year	3	9081,0	3027,01	998,35	0,000
Genoypte	2157	13813,3	6,40	2,11	0,000
Error	6462	19592,8	3,03		
Total	8625	63072,3			

Supplementary Table S2. The Spearman’s correlation coefficients between obligate pathogens in 1389 winter wheat genotypes for the period 2016-2019

		Powdery mildew*	Yellow rust*	Leaf rust*	Stem rust*
	Powdery mildew	n/a	r= 0.03 (*P*=0.330)	r= -0.07 (*P*=0.010)	n/a
2016	Yellow rust	n/a	n/a	**r= -0.18 (*P*<0.001)**	n/a
	Leaf rust	n/a	n/a	n/a	n/a
	Stem rust	n/a	n/a	n/a	n/a
	Powdery mildew	n/a	**r= 0.29 (*P*<0.001)**	r= 0.08 (*P*=0.005)	n/a
2017	Yellow rust	n/a	n/a	r= -0.10 (*P*<0.001)	n/a
	Leaf rust	n/a	n/a	n/a	n/a
	Stem rust	n/a	n/a	n/a	n/a
	Powdery mildew	n/a	r=-0.16 (*P*<0.001)	r= -0.05 (*P*=0.065)	n/a
2018	Yellow rust	n/a	n/a	**r= -0.43 (*P*<0.001)**	n/a
	Leaf rust	n/a	n/a	n/a	n/a
	Stem rust	n/a	n/a	n/a	n/a
	Powdery mildew	n/a	r=0.04 (*P*=0.181)	r= 0.04 (*P*=0.117)	n/a
2019	Yellow rust	n/a	n/a	r= -0.27 (*P*<0.001)	n/a
	Leaf rust	n/a	n/a	n/a	n/a
	Stem rust	**r= 0.28 (*P*<0.001)**	r= 0.03 (*P*=0.230)	r= -0.01 (*P*=0.600)	n/a

***** The relationship among obligate pathogens in each growing season was characterized on 1389 genotypes showing susceptibility to each obligate pathogen (disease index>30%) in at least one year in the period 2016-2019.

Supplementary Table S3. Regression analysis of the most influencing factors on disease indices of powdery mildew and yellow rust in 740 winter wheat genotypes in 2016 and 2018

**Set of genotypes where yellow rust predominated over powdery mildew in 2018**					
**Depenedent variable: disease index of yellow rust ***					
Source	DF	Adj SS	Adj MS	F-Value	P-Value
Regression	518	162041	312.8	1.62	0.000
T in January	1	18866	18866.2	97.85	0.000
Genotype	517	143070	276.7	1.44	0.000
Error	524	101036	192.8		
Total	1042	263077			
**Depenedent variable: disease index of powdery mildew ***					
Source	DF	Adj SS	Adj MS	F-Value	P-Value
Regression	518	93968	181.41	1.74	0.000
**T in January**	**1**	**1377**	**1377.13**	**13.19**	**0.000**
**Genotype**	**517**	**92588**	**179.09**	**1.71**	**0.000**
Error	524	54724	104.44		
Total	1042	148693			
**Set of genotypes where powdery mildew predominated over yellow rust in 2018**					
**Depenedent variable: disease index of yellow rust ***					
Source	DF	Adj SS	Adj MS	F-Value	P-Value
Regression	219	67483	308.141	1.84	0.000
Powdery mildew	1	816	816.109	4.87	0.028
T in January	1	665	665.044	3.97	0.048
Genotype	217	59724	275.225	1.64	0.000
Error	218	36518	167.515		
Total	437	104001			
**Depenedent variable: disease index of powdery mildew ***					
Source	DF	Adj SS	Adj MS	F-Value	P-Value
Regression	219	92318	421.5	3.50	0.000
**Yellow rust**	**1**	**586**	**586.1**	**4.87**	**0.028**
**T in February**	**1**	**43819**	**43818.8**	**364.26**	**0.000**
**Genotype**	**217**	**34217**	**157.7**	**1.31**	**0.023**
Error	26225	120.3	218		
Total	437	118542			

***** Climatic factors related to both growing seasons (2015/2016 and 2017/2018) were subjected to multiple linear regression together with effect of genotypes and competing obligate pathogens to investigate the most influential factors on the disease indices of yellow rust and powdery mildew in the two sets of genotypes Table of variances indicated the most influencing factors on disease indices of powdery mildew and yellow rust in two growing seasons and two sets of genotypes.

Supplementary Table S4. Regression analysis of the most influencing factors on disease indices of yellow rust and leaf rust in 303 winter wheat genotypes in 2016 and 2018

Set of genotypes where yellow rust predominated over leaf rust in 2018					
**Depenedent variable: disease index of yellow rust ***					
Source	DF	Adj SS	Adj MS	F-Value	P-Value
Regression	221	93281	422.1	2.09	0.000
T in January	1	10865	10865.2	53.88	0.000
Leaf rust	1	1189	1189.4	5.90	0.016
Genotype	219	83087	379.4	1.88	0.000
Error	220	44367	201.7		
Total	441	137648			
Depenedent variable: **disease index of leaf rust** *					
Source	DF	Adj SS	Adj MS	F-Value	P-Value
Regression	1	2041.9	2041.86	47.97	0.000
**T in January**	**1**	**2041.9**	**2041.86**	**47.97**	**0.000**
Error	440	18729.4	42.57		
Total	441	20771.3			
**Set of genotypes where leaf rust predominated yellow rust in 2018**					
**Depenedent variable: disease index of yellow rust ***					
Source	DF	Adj SS	Adj MS	F-Value	P-Value
Regression	1	12076.3	12076.3	45.10	0.000
Leaf rust	1	12076.3	12076.3	45.10	0.000
Error	162	43382.0	267.8		
Total	163	55458.3			
**Depenedent variable: disease index of leaf rust***					
Source	DF	Adj SS	Adj MS	F-Value	P-Value
Regression	2	90627	45313.3	507.07	0.000
**T in February**	**1**	**67759**	**67759.3**	**758.25**	**0.000**
**Yellow rust**	**1**	**948**	**948.3**	**10.61**	**0.001**
Error	161	14387	89.4		
Total	163	105014			

***** Climatic factors related to both growing seasons (2015/2016 and 2017/2018) were subjected to multiple linear regression together with effect of genotypes and competing obligate pathogens to investigate the most influential factors on the disease indices of yellow rust and leaf rust in the two sets of genotypes Table of variances indicated the most influencing factors on disease indices of leaf rust and yellow rust in two growing seasons and two sets of genotypes.

Supplementary Table S5. Regression analysis of the most influencing factors on occurrence of stem rust in phenotyping platform in 2019

Source	DF	Adj SS	Adj MS	F-Value	P-Value
Regression	3	5098,78	1699,59	536,15	0,000
Powdery mildew	1	34,73	34,73	10,96	0,001
Leaf rust	1	22,95	22,95	7,24	0,007
Total rainfall in November	1	2928,35	2928,35	923,76	0,000
Error	1521	4821,59	3,17		
Total	1524	9920,37			
