# Supplementary material for: Diversity in susceptibility reactions of winter wheat genotypes to obligate pathogens under fluctuating climatic conditions
Source: Sci Rep. 2020 Nov 12;10:19608. doi: 10.1038/s41598-020-76693-z (PMC7665191; doi:10.1038/s41598-020-76693-z)
Supplement: Supplementary file 8 — Supplementary Captions. [file 41598_2020_76693_MOESM8_ESM.docx]

**Supplementary Figure S1.** Residual analysis of multiple regression on the factors influencing disease indices of powdery mildew in the set of genotypes where yellow rust predominated powdery mildew in 2016 and powdery mildew predominated yellow rust in 2018

**Supplementary Figure S2.** Average temperatures and total rainfall in locality Rimski Šančevi in the period 2016-2019

**Supplementary Table S1.** Regression analysis of the most influencing factors on disease indices of obligate pathogens in 2158 winter wheat genotypes for the period 2016-2019

**Supplementary Table S2.** The Spearman’s correlation coefficients between obligate pathogens in 1389 winter wheat genotypes for the period 2016-2019

* The relationship among obligate pathogens in each growing season was characterized on 1389 genotypes showing susceptibility to each obligate pathogen (disease index˃30%) in at least one year in the period 2016-2019.

**Supplementary Table S3.** Regression analysis of the most influencing factors on disease indices of powdery mildew and yellow rust in 740 winter wheat genotypes in 2016 and 2018

***** Climatic factors related to both growing seasons (2015/2016 and 2017/2018) were subjected to multiple linear regression together with effect of genotypes and competing obligate pathogens to investigate the most influential factors on the disease indices of yellow rust and powdery mildew in the two sets of genotypes Table of variances indicated the most influental factors on disease indices of powdery mildew and yellow rust in two growing seasons and two sets of genotypes.

**Supplementary Table S4.** Regression analysis of the most influencing factors on disease indices of yellow rust and leaf rust in 303 winter wheat genotypes in 2016 and 2018

***** Climatic factors related to both growing seasons (2015/2016 and 2017/2018) were subjected to multiple linear regression together with effect of genotypes and competing obligate pathogens to investigate the most influential factors on the disease indices of yellow rust and leaf rust in the two sets of genotypes Table of variances indicated the most influential factors on disease indices of leaf rust and yellow rust in two growing seasons and two sets of genotypes.

**Supplementary Table S5.** Regression analysis of the most influencing factors on occurrence of stem rust in phenotyping platform in 2019

**Supplementary Dataset 1.** Winter wheat genotypes included in phenotyping platform

*Dataset includes 2158 winter wheat genotypes, DI of obligate pathogens assessed in 2016-2019 and pedigrees.

**Supplementary Dataset 2.** Winter wheat genotypes showing susceptibility to each obligate pathogen in at least one year in the period 2016-2019

*Dataset includes 1389 winter wheat genotypes with DI exceeding 30% to each obligate pathogen in at least one year in the period 2016-2019.

**Supplementary Dataset 3.** Winter wheat genotypes included in analysis of the inconsistency in the predominance of powdery mildew and yellow rust in the same field and growing season

*Dataset includes 740 genotypes divided into two sets: 1) powdery mildew predominated over yellow rust in 2018, and 2) yellow rust predominated over powdery mildew in 2018. Dataset also shows DI of obligate pathogens assessed in 2016 and 2018 and pedigrees.

**Supplementary Dataset 4.** Winter wheat genotypes included in analysis of the inconsistency in the predominance of leaf rust and yellow rust in the same field and growing season

*Dataset includes 303 genotypes divided into two sets: 1) leaf rust predominated over yellow rust in 2018, and 2) yellow rust predominated over leaf rust in 2018. Dataset also shows DI of obligate pathogens assessed in 2016 and 2018 and pedigrees.

**Supplementary Dataset 5.** Occurrence of stem rust in the phenotyping platform in 2019

*Dataset includes 2158 winter wheat genotypes and DI assessments of stem rust in 2019.
